# Composition and Bioactivity of a Modified Huang-Lian-Jie-Du Decoction

**DOI:** 10.1155/2022/2147923

**Published:** 2022-09-27

**Authors:** Mei-Yi Lin, Lih-Geeng Chen, Ying-Yu Siao, Tao-Hsuan Lin, I-An Huang, Yi-Wen Liu, Chin-Chin Huang

**Affiliations:** ^1^Department of Chinese Medicine, Ditmanson Medical Foundation Chiayi Christian Hospital, Chiayi 600, Taiwan; ^2^Department of Food Nutrition and Health Biotechnology, Asian University, Taichung 41354, Taiwan; ^3^Department of Microbiology, Immunology and Biopharmaceuticals, College of Life Sciences, National Chiayi University, Chiayi 600, Taiwan

## Abstract

**Background:**

Epidermal growth factor receptor inhibitors (EGFRIs) and tyrosine kinase inhibitors (TKIs) are key drugs in targeted cancer therapy. However, they may cause skin toxicity. We previously prepared a modified Huang-Lian-Jie-Du (mHLJD) decoction cream using 10 herbs, which effectively alleviated EGFRI/TKI-induced skin toxicity. In the present study, we identified the reference markers of the mHLJD decoction and investigated the anti-inflammatory and antibacterial effects of the mHLJD decoction extract.

**Methods:**

We performed high-performance liquid chromatography (HPLC) to determine the composition of the mHLJD decoction. Human epidermoid A431 cells were treated with tumor necrosis factor (TNF)-*α* to induce inflammation; then, the effects of the mHLJD decoction extract on the cytokine expression were determined using a cytokine array and by performing real-time quantitative polymerase chain reaction (qPCR). The antibacterial effects of the extract were examined using disk diffusion and microdilution assays.

**Results:**

HPLC results revealed that the mHLJD decoction primarily consisted of geniposide, berberine chloride, baicalin, coptisine, and palmatine. TNF-*α* treatment increased the expression of certain cytokines, including IL-1*β*, IL-8, M-CSF, and TGF-*β*2; however, pretreatment with the mHLJD decoction extract reduced their expression. The qPCR results demonstrated a decreased mRNA expression of IL-8, M-CSF, and TGF-*β*2. The antibacterial assay revealed that the extract exerted inhibitory effects on *Staphylococcus aureus*, forming an inhibition zone at the minimum inhibitory concentrations of 3.125 and 6.25 mg/mL; however, the extract exerted no effects on *Escherichia coli* and *Pseudomonas aeruginosa. Conclusions*. We developed an HPLC method to quantify the reference markers of the mHLJD decoction. The bioactivity analysis provided the potential mechanisms underlying the effects of the mHLJD decoction on EGFRI/TKI-induced skin toxicity.

## 1. Introduction

The Huang-Lian-Jie-Du (HLJD) decoction is a traditional Chinese medicine formula with a long history of use for the treatment of various diseases. This decoction consists of four traditional herbs, namely *Huanglian* (*Coptidis Rhizoma*), *Huangbo* (*Phellodendri Cortex*), *Huangqin* (*Scutellariae Radix*), and *Zhizi* (*Gardeniae Fructus*), with a dry weight ratio of 3 : 2 : 2 : 3, respectively [1]. The HLJD decoction has been used to treat tumors, hepatic diseases, inflammation, allergies, blood lipid and glucose disorders, central nervous system diseases, bacterial infections, and intestinal flora disturbances [2]. In our previous study, we prepared a modified HLJD (mHLJD) decoction cream and used it to treat adverse skin effects caused by epidermal growth factor receptor inhibitors (EGFRIs)/tyrosine kinase inhibitors (TKIs) [3]. In addition to the four aforementioned herbs, the following six herbs were added to enhance the efficacy of the traditional decoction: *Sangbaipi* (*Mori Cortex*), *Longdan* (*Gentianae Radix et Rhizoma*), *Kushen* (*Sophorae flavescentis Radix*), *Tofulin (Smilacis Glabrae Rhizoma), Lianqiao* (*Forsythiae Fructus*), and *Shigao* (*Gypsum Fibrosum*). These additional herbs were selected because they can effectively clear heat and remove moisture. Traditionally, the HLJD decoction is administered orally that phytochemicals are absorbed from the gut and exert systemic effects on the body. However, our mHLJD decoction was formulated as a cream for topical use to minimize systemic effects. The cream safely and effectively improved EGFRI/TKI-induced acneiform rash and dry skin [3].

The major phytochemical constituents of the four herbal medicines included in the traditional HLJD decoction include berberine (*Huanglian* and *Huangbo*), baicalin and baicalein (*Huangqin*), and geniposide (*Zhizi*). In the present study, we determined the constituents of the mHLJD decoction. According to *TaiwanHerbal Pharmacopeia*, *Third Edition*, the reference markers of the following herbs are as follows: *Huanglian*, berberine >4.2%; Huangbo, berberine >1.2%; *Huangqin*, baicalin >8.0%; and *Zhizi*, geniposide >1.8%. The reference markers of the six additional herbs are as follows: *Tofulin*, astilbin >0.45%; *Kushen*, matrine and oxymatrine >1.2%; and *Longdan*, gentiopicrin >3.0%. *Lianqiao* has green and old forms, each with different contents of reference markers: green *Lianqiao*, phillyrin >0.30%, and forsythoside A >2.0%; old *Lianqiao*, phillyrin >0.09%, and forsythoside A >0.25% [4].

Although our knowledge regarding the mechanisms underlying EGFRI/TKI-induced skin toxicity remains limited, studies thus far have provided some insights. Mice lacking epidermal EGFR exhibited chronic skin inflammation similar to that induced by EGFRIs/TKIs in humans, including the infiltration of macrophages and the expression of cytokines (e.g., interleukin (IL)-1*β*, C-C chemokine receptor type 5, and tumor necrotic factor (TNF)-*α*) [5]. In multiple lung cancer cell lines, EGFR signaling effectively reduced the levels of TNF-*α*, and the inhibition of EGFR resulted in the increased levels of TNF-*α* through the alteration of mRNA stability [6]. Therefore, to evaluate the anti-inflammatory effects of the mHLJD decoction in the present study, TNF-*α* was used to establish an in vitro model of skin inflammation in the human skin cell line A431.

EGFRI/TKI-induced skin toxicity poses an increased risk of microbial infection. Bacterial skin and soft tissue infections (SSTIs) are common in the community and hospitals. Various bacteria can cause SSTIs. Among them, infections caused by Gram-positive bacteria are predominant, particularly those caused by *Staphylococcus aureus*, which is the most common pathogen present on the human skin [7–9]. In addition, *Pseudomonas aeruginosa* causes fatal nosocomial infections in patients admitted to the intensive care unit and immunocompromised patients. Furthermore, *Escherichia coli* and other Enterobacteriaceae are common in chronic wound infections and are primarily responsible for Gram-negative bacterial SSTIs [9]. Therefore, we used these bacteria in the present study to evaluate the antibacterial effects of the mHLJD decoction.

The mHLJD decoction is a new formulation. To the best of our knowledge, this decoction has been used as a cream formulation for the first time. This cream formulation must be explored to elucidate mechanism underlying its beneficial effects. Thus, this study investigated mechanisms through which the mHLJD decoction alleviates skin symptoms. The findings of this study can facilitate drug development in the future. In addition, this study identified the reference markers of the mHLJD decoction and elucidated mechanisms underlying the anti-inflammatory and antibacterial effects of this decoction. Our findings may facilitate the clinical application of the mHLJD decoction cream for patients with EGFRI/TKI-induced skin toxicity.

## 2. Materials and Methods

### 2.1. Reagents for High-Performance Liquid Chromatography

Berberine, coptisine, palmatine, forsythoside A, phillyrin, astibin, baicalin, geniposide, and gentiopicrin (reference markers for high-performance liquid chromatography (HPLC)) were purchased from Fusol Material Co., Ltd. (Tainan, Taiwan). Acetonitrile and methanol were obtained from Spectrum Chemical MFG. Corp. (HPLC grade; New Brunswick, NJ, USA). Monopotassium phosphate and sodium lauryl sulfate were purchased from J. T. Baker (Philipsburg, NJ, USA). Trifluoroacetic acid (purity >99.5%; HPLC grade) was obtained from Alfa Aosor (Lancashire, UK).

### 2.2. Preparation of the mHLJD Decoction Extract for Bioactivity Analysis

A total of 10 herbs, namely *Huanglian*, *Huangbo*, *Huangqin*, *Zhizi*, *Sangbaipi*, *Kushen*, *Longdan*, *Lianqiao*, *Tofulin*, and *Shigao*, were required to prepare the mHLJD decoction. These herbs were purchased from Fu-Xian herbal medicine store (Chiayi, Taiwan) and were identified by a professional pharmacist, I-An Huang, at Ditmanson Medical Foundation Chia-Yi Christian Hospital, Taiwan. The specimens of these 10 herbs have been deposited at the Department of Chinese Medicine of the same hospital. The mHLJD decoction was prepared through hot water extraction as previously reported [3]. The decoction was then freeze-dried to obtain the mHLJD decoction extract. For bioactivity analysis, the freeze-dried mHLJD powder was mixed with a serum-free culture medium (50 mg/mL) and subsequently sonicated on ice for 5 s (four times). The suspension was then centrifuged at 850 × g for 15 min at 8°C. The supernatant was filtered using qualitative Millex filter papers (0.2 and 0.45 *μ*m; Merck Millipore, Darmstadt, Germany) and stored at −80°C until use. The stock concentration of the mHLJD decoction extract was 50 mg/mL, and dilutions were prepared in a culture medium.

### 2.3. Reference Markers of the mHLJD Decoction Analyzed Using HPLC

For HPLC, we used the Shimadzu LC-10ATvp liquid chromatograph with DGU-14ALvp, DGU-14A degasser, SIL-10ADvp auto injector, SCL-10Avp system controller, SPD-M10Avp diode array detector, and LCsolution ver. 1.25 software (Shimadzu, Kyoto, Japan). The Ascentis Express 90A C18 column (4.6 mm × 25 cm; 5 *µ*m; Supelco) was used for the HPLC analysis of gentiopicrin, geniposide, astilbin, berberine chloride, and baicalin. The mobile phase consisted of water and 0.05% trifluoroacetic acid–acetonitrile–methanol under the following gradient program: 0–55 min, 90 : 5:5 ⟶ 50 : 5:45 linear gradient; 55–56 min, 50 : 5:45 ⟶ 0 : 0:100 linear gradient; 56–60 min, 0 : 0:100 isocratic; 60–61 min, 0 : 0:100 ⟶ 90 : 5:5 linear gradient; and 61–65 min, 90 : 5:5 isocratic. The flow rate was 1.0 mL/min, and 10 *μ*L was injected into the column. The column temperature was maintained at 40°C. Chromatograms were recorded at 230 nm.

Coptisine, palmatine, and berberine chloride were determined following the assay method recommended in *Taiwan Herbal Pharmacopeia*, *Third Edition*, by using the Purospher STAR RP-18e column: elution buffer, 45 : 55 acetonitrile:H_2_O (containing monopotassium phosphate, 3.4 g/L and sodium lauryl sulfate, 1.7 g/L); flow rate, 1.0 mL/min; column temperature, 40°C; and detection wavelength, 345 nm.

### 2.4. Preparation of Calibration Curves for HPLC

Ten milligrams of gentiopicrin (C_16_H_20_O_9_; molecular weight (MW), 356.32 g/mol), geniposide (C_17_H_24_O_10_; MW, 388.4 g/mol), astilbin (C_21_H_22_O_11_; MW, 450.4 g/mol), berberine chloride (C_20_H_18_ClNO_4_; MW, 371.8 g/mol), baicalin (C_21_H_18_O_11_; MW, 446.4 g/mol), coptisine chloride (C_19_H_14_ClNO_4_; MW, 355.8 g/mol), and palmatine chloride (C_21_H_22_ClNO_4_; MW, 387.9 g/mol) were added into separate volumetric flasks and dissolved in 10 mL of HPLC-grade methanol to prepare 1 mg/mL stock solutions. The stock solutions were serially diluted from 200 to 3.125 *μ*g/mL and then subjected to HPLC. The calibration curve (*y* = *ax* + *b*) was prepared using linear regression with the marker concentration as the *X* axis and the peak area as the *Y* axis.

### 2.5. Cell Culture and Treatment

Human epidermoid carcinoma A431 cells were purchased from Bioresource Collection and Research Center ((BCRC) Hsinchu, Taiwan) and maintained in Dulbecco's modified Eagle medium (Invitrogen, Carlsbad, CA, USA) supplemented with 10% fetal bovine serum, 1% penicillin, and 1% streptomycin. The cells were incubated at 37°C under 5% CO_2_ and 95% filtered air. The mHLJD decoction extract was added into the medium 1 h before TNF-*α* treatment.

### 2.6. Cell Viability Assay

Cell viability was determined using the 3-(4, 5-dimethylthiazol-2-yl)-2, 5-diphenyltetrazolium bromide (MTT) assay. A431 cells were cultured in 24-well plates (6 × 10^4^ cells/well) for 24 h and then incubated with various concentrations of the mHLJD decoction extract for 24 h. MTT (stock solution, 50 mg/mL; amount, 25 *µ*L; AK Scientific, Union City, CA, USA) was added to each well, and the plates were incubated for 1 h at 37°C under 5% CO_2_. After incubation, the medium was discarded and 500 *µ*L of dimethyl sulfoxide was added to each well and mixed using a micropipette. Then, the suspension (100 *µ*L) was transferred to a 96-well plate; absorbance was recorded at 595 nm.

### 2.7. Cytokine Array Analysis

After the indicated treatment, the cell culture medium was collected and analyzed for changes in the levels of various cytokines by using Human Cytokine Array C5 (RayBiotech, Peachtree Corners, GA, USA) following the manufacturer's instructions.

### 2.8. Real-Time Quantitative Polymerase Chain Reaction

Total RNA was extracted from the A431 cells by using TRIzol reagent (Invitrogen). For reverse transcriptase reactions, 1 *μ*g of RNA was used to synthesize complementary (c)DNA by using a reverse transcription kit (Invitrogen). The resulting cDNA was used to detect the expression of specific mRNAs through real-time quantitative polymerase chain reaction (qPCR) with SYBR Green by using the StepOne real-time PCR system (Applied Biosystems, Carlsbad, CA, USA). The following gene-specific primers were used:

- Human IL-8 (248 bp): forward primer, TTggCAgCCTTCCTgATTTC and reverse primer, AACTTCTCCACAACCCTCTgCA
- Human transforming growth factor beta 2 (TGF-*β*2; 146 bp): forward primer, CAgTgggAAgACCCCACATC and reverse primer, AAAgTggACgTAggCAgCAA
- Human glyceraldehyde 3-phosphate dehydrogenase (GAPDH; 496 bp): forward primer, CAAggTCATCCATgACAACTTTg and reverse primer, gTCCACCACCCTgTTgCTgTAg
- Human IL-1β (144 bp): forward primer, TCgCCAgTgAAATgATggCT and reverse primer, ggTCggAgATTCgTAgCTgg
- Human macrophage colony-stimulating factor (M-CSF; 124 bp): forward primer, gAACTgCCAgTgTAgAgggAAT and reverse primer, gCTggTCAgACAACATCTgg

After cDNA denaturation at 95°C for 10 min, 40 cycles of qPCR were performed; each cycle comprised denaturation at 95°C for 15 s, followed by annealing and extension at 60°C for 1 min. The relative gene expression was analyzed using the 2^−∆∆Ct^ method, with GAPDH RNA as the reference.

### 2.9. Microorganisms

The antimicrobial effects of the mHLJD decoction extract were evaluated using the disk diffusion test; a microdilution assay was performed to determine the minimum inhibitory concentration (MIC). Two Gram-negative bacterial strains (*E. coli* ATCC 8739 and *P. aeruginosa* ATCC 9027) and three Gram-positive bacterial strains (*S. aureus* ATCC 6538, 25923, and 29213) were purchased from the BCRC. All the bacterial strains were grown on Luria–Bertani agar plates. Overnight cultures were used to evaluate the antimicrobial activity of the extract. Fresh colonies were suspended in Mueller–Hinton (MH) broth and incubated at 37°C for 2 h immediately before use.

### 2.10. Disk Diffusion Test

A modified disc diffusion test was performed to detect the antimicrobial activity of the mHLJD decoction extract [10]. First, MH agar was poured into 90-mm Petri dishes until it reached a mean depth of 4.0 ± 0.5 mm. Then, 5 mg of test samples were loaded on 6-mm Oxoid antimicrobial susceptibility test disks. The disks were left to dry overnight at 30°C under sterile conditions. The density of the test bacterial cultures was adjusted to 0.5 McFarland standard (approximately 10^8^ colony-forming units (CFU)/mL). Bacterial suspensions were streaked on the surface of the agar plates and then left to dry for 5 min at room temperature. Subsequently, the test sample-loaded disks were added firmly onto the surface of the plates. Finally, the plates were incubated overnight at 37°C. Inhibition diameters (mm) were measured after 24 h.

### 2.11. Microdilution Assay

The MICs of the test samples were determined using a modified version of the methods described by Fawole et al. [11] and Kuo et al. [12]. The assay was performed using 96-well microtiter plates. The test bacteria were suspended in the MH broth to ensure a density of 0.5 standard McFarland and then diluted (10 folds) twice to ensure a density of approximately 10^6^ CFU/mL. The microplate wells were inoculated with 100 *μ*L of the diluted suspensions. The test samples (100 *μ*L) were serially diluted (two folds) in the MH broth to obtain a total of 10 concentrations ranging from 0.049 to 25 mg/mL. Streptomycin (diluted from 100 to 0.195 *μ*g/mL) was used as a positive control, whereas bacteria-free MH broth was used as a negative control. The tests were performed in triplicate; the plates were incubated at 37°C for 18–24 h. Subsequently, the microplate wells were examined under the unaided eye for bacterial growth (turbidity). The last concentration in the dilution series of a test sample that did not exhibit visible growth was considered to the MIC of the sample.

### 2.12. Statistical Analysis

Data were presented as mean ± SEM. One-way analysis of variance with Tukey's multiple comparison test was performed for statistical analyses. Significant differences were marked as ^*∗*^*p* < 0.05, ^*∗∗*^*p* < 0.01, and ^*∗∗∗*^*p* < 0.001.

## 3. Results

### 3.1. Reference Markers of the mHLJD Decoction

**Figure 1:**
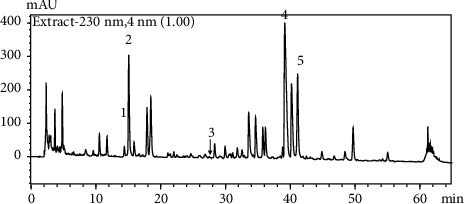
The HPLC chromatogram of the mHLJD decoction. Column: Ascentis Express 90A C18 (4.6 mm × 25 cm; 5 *µ*m; Supelco); mobile phase: 0.05% trifluoroacetic acid–acetonitrile–methanol (0 min, 90 : 5:5; 55 min, 50 : 5 : 45; 56 min, 0 : 0 : 100; 60 min, 0 : 0 : 100; 61 min, 90 : 5 : 5; and 65 min, 90 : 5 : 5); flow rate: 1.0 mL/min; recorded at 230 nm; column temperature maintained at 40°C. 1: gentiopicrin; 2: geniposide; 3: astilbin; 4: berberine chloride; and 5: baicalin.

**Figure 2:**
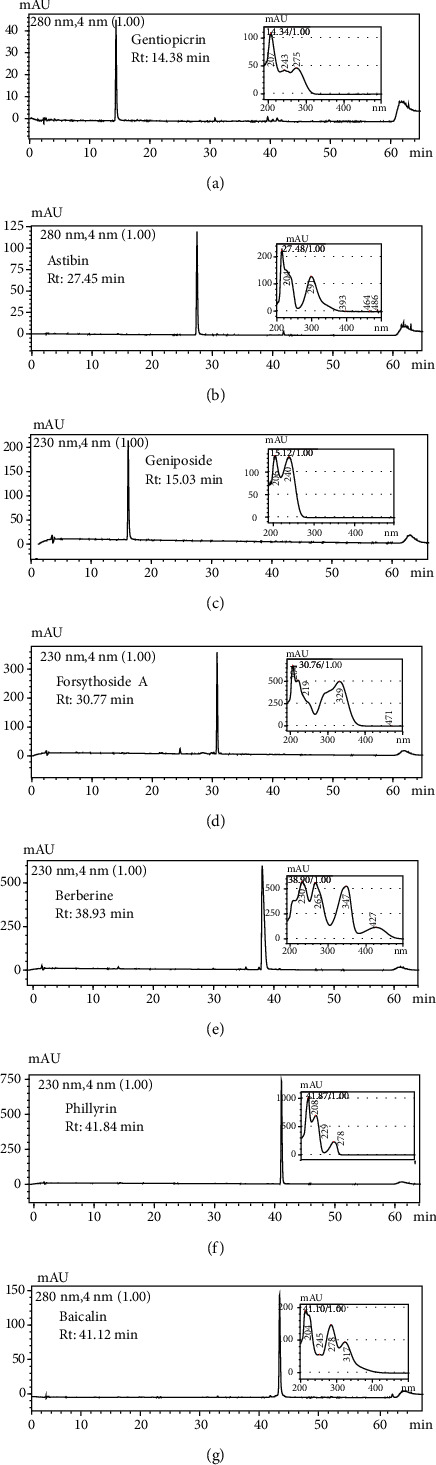
The chromatograms of seven reference markers: (a) gentiopicrin; (b) astilbin; (c) geniposide; (d) forsythoside A; (e) berberine chloride; (f) phillyrin; and (g) baicalin. Column: Ascentis Express 90A C18 (4.6 mm × 25 cm; 5 *µ*m; Supelco); mobile phase: 0.05% trifluoroacetic acid–acetonitrile–methanol (0 min, 90 : 5 : 5; 55 min, 50 : 5 : 45; 56 min, 0 : 0 : 100; 60 min, 0 : 0 : 100; 61 min, 90 : 5 : 5; and 65 min, 90 : 5 : 5); flow rate: 1.0 mL/min; column temperature maintained at 40°C. Chromatograms were recorded at 230 nm for geniposide, berberine chloride, phillyrin, and forsythoside A, and 280 nm for gentipicrin, astilbin, and baicalin.

**Figure 3:**
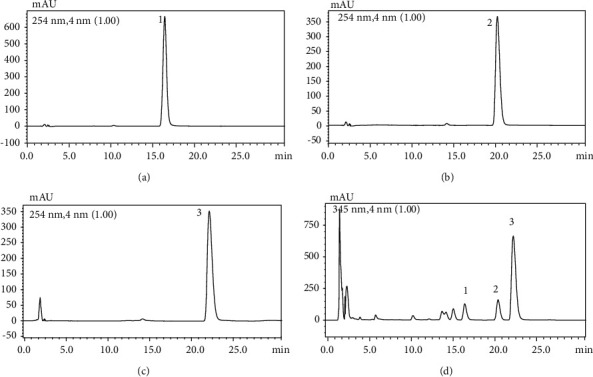
The HPLC chromatograms of (a) coptisine chloride, (b) palmatine chloride, (c) berberine chloride, and (d) mHLJD decoction extract. Column: Purospher STAR RP-18e column; mobile phase, 45 : 55 acetonitrile:H_2_O (contained monopotassium phosphate, 3.4 g/L and sodium lauryl sulfate, 1.7 g/L); flow rate, 1.0 mL/min; recorded at 345 nm; column temperature maintained at 40°C. 1: coptisine chloride; 2: palmatine chloride; and 3: berberine chloride.

The quantitative analysis of the mHLJD decoction consisting of the 10 herbs through HPLC was difficult. First, we set the parameters for HPLC. The Purospher STAR RP-18e column (4 mm (internal diameter) × 250 mm; 5 *μ*m; Merck) could not ensure satisfactory peak separation and resolution (data not shown). The Ascentis Express 90A C18 column (4.6 mm × 25 cm; 5 *µ*m; Supelco) is a core-shell-type column with a narrow peak width and resolution. Furthermore, after mobile phase optimization, we used a 0.05% trifluoroacetic acid-acetonitrile-methanol gradient to ensure the best condition for separation (Figure 1). A total of seven reference markers could be separated in this condition (Figure 2). However, only gentiopicrin, geniposide, astilbin, berberine, and baicalin were detected in this condition, as confirmed using data obtained with the photodiode array detector. The reference markers of *Lianqiao*—forsythoside A and phillyrin—could not be detected in the decoction.  Table 1 presents the regression equation of the five reference markers of the mHLJD decoction. The contents of gentiopicrin, geniposide, astilbin, berberine chloride, and baicalin in the lyophilized mHLJD decoction were 1.37, 6.15, 0.01, 3.06, and 6.85 mg/g, respectively. Coptisine chloride, palmatine chloride, and berberine chloride were determined using the assay method recommended in *Taiwan Herbal Pharmacopeia*, *Third Edition*, by using the Purospher STAR RP-18e column (Figure 3). Because the peak resolution shown in Figure 3 is better than that shown in Figure 2, the relevant results appear to be more precise. The content of coptisine, palmatine, and berberine chloride in the lyophilized mHLJD decoction were 7.42, 17.05, and 3.57 mg/g, respectively.

### 3.2. Cytotoxicity of the mHLJD Decoction Extract to A431 Cells

**Figure 4:**
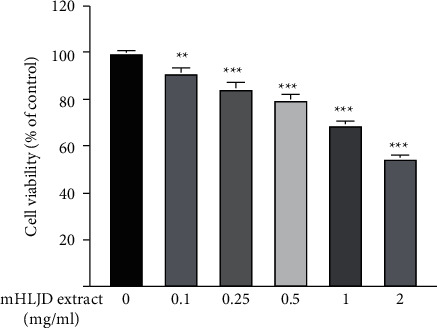
Cytotoxicity of the mHLJD decoction extract to A431 cells. A431 cells were cultured in 24-well plates (6 × 10^4^ cells/well) for 24 h and then incubated with the mHLJD decoction extract for 24 h and cell viability was calculated using 100% as the control. ^*∗∗*^*p* < 0.01 and ^*∗∗∗*^*p* < 0.001 compared with the control group.

The cytotoxicity of the mHLJD decoction extract to the A431 cells was examined to determine a nontoxic dose for bioactivity analysis. After 24 h of treatment with various concentrations of the mHLJD decoction extract, we determined a decrease in cell viability with the increasing concentration of the extract (Figure 4). The survival rate of the cells pretreated with 0.5 mg/mL of the extract was approximately 80%; thus, this concentration was used in the subsequent experiments.

### 3.3. TNF-*α* and the mHLJD Decoction Extract Altered the Cytokine Expression

**Figure 5:**
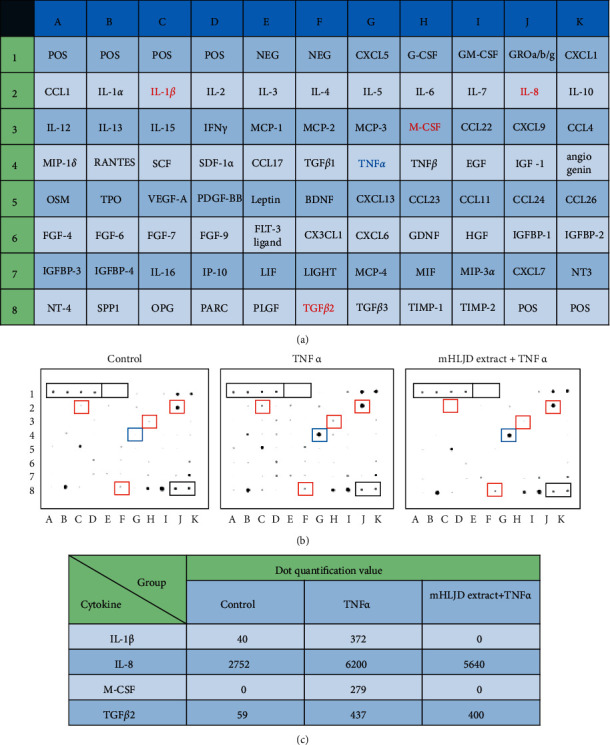
Effects of TNF-*α* and the mHLJD decoction extract on cytokine production in A431 cells. (a) The cytokine name of each dot on the Human Cytokine Array C5. POS: positive control spot and NEG: negative control spot. (b) Array results of culture medium from control, TNF-*α*, and mHLJD plus TNF-*α* groups. A431 cells were incubated with (TNF-*α* group and mHLJD extract plus TNF-*α* group) or without (control) 25 ng/mL TNF-*α* for 6 h. In the mHLJD extract plus TNF-*α* group, 0.5 mg/mL of the mHLJD decoction extract was added 1 h before TNF-*α* treatment. The culture medium was collected for blotting with the array membrane. Black frame: positive and negative control spot; blue frame: TNF-*α*; and red frame: spots of IL-1*β* (C2), IL-8 (J2), M-CSF (H3), and TGF-*β*2 (F8). (c) The normalized quantification values of 12 dots including 4 cytokines in 3 groups.

The effects of TNF-*α* on the cytokine production in the A431 cells was analyzed using a cytokine array assay. The hybridization membrane had 80 cytokine spots, with 6 positive and 2 negative control spots. As presented in Figure 5, the TNF-*α* increased the protein levels of IL-1, IL-8, M-CSF, and TGF-*β*2, whereas the mHLJD decoction extract pretreatment reduced these levels. The levels of TNF-*α* were increased because of its external addition.

### 3.4. Effects of mHLJD Decoction Extract on the mRNA Expression of IL-1*β*, IL-8, M-CSF, and TGF-*β*2 in A431 Cells

**Figure 6:**
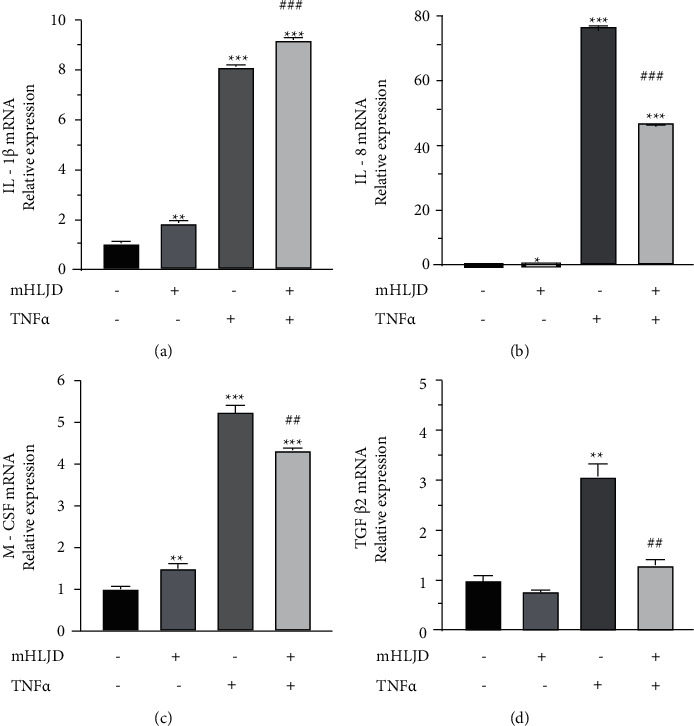
Effects of the mHLJD decoction extract on TNF-*α*-induced IL-1*β*, IL-8, M-CSF, and TGF-*β* 2 gene expression in A431 cells. The relative mRNA expression was quantitatively detected by the StepOne real-time PCR system using the 2−^∆∆Ct^ method with GAPDH RNA as a reference. ^*∗*^*p* < 0.05 and ^*∗∗∗*^*p* < 0.001 compared with the control group. ^###^*p* < 0.001 compared with the TNF-*α* and group.

Because the protein expression of IL-1*β*, IL-8, M-CSF, and TGF-*β*2 in the A431 cells was increased after treatment with TNF-*α* but decreased after pretreatment with the mHLJD decoction extract, we performed qPCR to assess their mRNA expression. As shown in Figure 6, the mHLJD decoction extract reduced the TNF-*α*-induced increases in the expression of IL-8, M-CSF, and TGF-*β*2 but not that in the expression of IL-1*β*. The IL-1*β* protein, but not its mRNA, was inhibited by the extract. This finding suggests that the mHLJD decoction extract inhibits the translation of IL-1*β* or increase the degradation rate of this protein. Among the four cytokines, TNF-*α* induced the highest increase in the expression of IL-8 and the lowest increase in that of TGF-*β*2. By contrast, the mHLJD decoction extract the largest effect on the expression of TGF-*β*2 and the smallest effect on that of M-CSF.

### 3.5. Antimicrobial Effects of the mHLJD Decoction Extract

The antibacterial effects of the mHLJD decoction extract were evaluated using disk diffusion and microdilution assays with various bacterial strains. The extract inhibited the growth of the three *S. aureus* strains; the diameters of the inhibition zones ranged from 9 to 10.33 mm. However, no inhibitory effects of the extract were noted against *E. coli* and *P. aeruginosa*. Similar results were obtained in the microdilution assay. *E. coli* and *P. * were resistant to even the highest test sample concentration of 25 mg/mL. All tested strains of *S. aureus* were sensitive to the mHLJD decoction extract, and the MICs ranged from 3.125 to 6.25 mg/mL (Table 2). These results substantiate the antibacterial effects of the mHLJD decoction extract on the Gram-positive bacteria *S. aureus* but not on the Gram-negative bacteria used in this study.

## 4. Discussion

We added a total of six additional herbs to the traditional HLJD decoction. *Taiwan Herbal Pharmacopeia*, *Third Edition*, does not include data regarding the reference markers of Shigao and Sangbaipi. Therefore, in the present study, we analyzed seven phytochemicals present in the mHLJD decoction. Furthermore, *Taiwan Herbal Pharmacopeia*, *Third Edition*, recommends using the best extraction solvent to identify the reference markers of herbal medicines; thus, we used water because water is safer than organic solvents and is used to prepare the traditional HLJD decoction. During the preparation of the decoction, the components may exhibit complex interactions and chemical modifications, which may lead to changes in their contents and levels. To the best of our knowledge, no appropriate HPLC detection method has been developed yet for the mHLJD decoction. Hence, in the present study, we developed an HPLC method to quantify the seven reference markers of the mHLJD decoction. The results of the chemical analysis indicated that the decoction primarily comprised geniposide, berberine chloride, baicalin, coptisine, and palmatine, all of which are the components of the traditional HLJD decoction. Coptisine is present in *Huanglian*, and palmatine is present in *Huanglian* and *Huangbo* [13, 14]. Coptisine, palmatine, and berberine are protoberberine alkaloids and have various pharmacological functions, including antibacterial and anti-inflammatory activities [15, 16].

TNF-*α* is a proinflammatory cytokine and is mainly produced by macrophages. For EGFR inhibition, the populations of dermal immune cells primarily include macrophages and mast cells; TNF-*α* secreted by macrophages promotes subsequent epidermal barrier defects and bacterial infections [17]. TNF-*α* stimulates various cells by binding to its specific receptors on the cells to promote several pathological pathways, such as those involved in rheumatoid arthritis, psoriasis, and cancer. In the present study, we examined the effects of TNF-*α* on epidermal cells. TNF-*α* stimulated the A431 cells to secrete some inflammatory agents, including IL-1*β*, IL-8, M-CSF, and TGF-*β*2. The expression of all the aforementioned agents was induced by TNF-*α*; however, the mHLJD decoction extract inhibited the expression of only IL-8, M-CSF, and TGF-*β*2. IL-8 is a chemokine that guides neutrophils to inflammatory sites [18]. The reduction of the IL-8 level with the IL-8 neutralizing antibody ameliorated EGFRI-induced dermatological side effects [19]. The mHLJD decoction extract reduced the TNF*α*-induced expression of IL-8, which may exert positive effects on EGFRI-induced acneiform rash [3].

Berberine, baicalin, and baicalein are the key phytochemicals in the herbal constituents of HLJD. Berberine and baicalin may be used to treat chronic respiratory disease involving lung inflammation [20]. Baicalin exhibits effective activities against coronavirus disease 2019 by activating the angiotensin-converting enzyme 2/angiotensin/Mas axis [20]. Baicalin and baicalein are flavonoids. Narirutin, a dietary flavonoid, exerts anti-inflammatory and immunomodulation effects [21]. The mHLJD decoction extract exhibits antibacterial activity, including inhibitory effects against *S. aureus*. Berberine, an isoquinoline alkaloid, exhibits anti-methicillin-resistant *S. aureus* activity by damaging the bacterial cell surface and releasing the intracellular content [22]. In addition to bacterial growth suppressing, baicalin and baicalein inhibit biofilm formation and attenuate the virulence of *S. aureus* [23, 24, 25]. These findings explain the anti-*S. aureus* activity of the mHLJD decoction extract in the present study. In addition to infection, microorganisms play other roles in skin diseases. The acute phase of atopic dermatitis is associated with changes in the skin microbiome: the decrease in bacterial diversity and the dominance of a single pathogen, *S. aureus*. *S. aureus* exacerbates the inflammation and further weakens the epidermal barrier. The dysbiosis of the skin microbiome should be improved to treat skin diseases [26]. To the best of our knowledge, whether skin microbiome dysbiosis and *S. aureus* are associated with EGFRI/TKI-induced skin toxicity remains unknown. This represents a topic for future studies on microbial pathogenesis and relevant treatment.

Berberine has various pharmacological roles, including anti-inflammatory and antimicrobial activities [27]. Wu et al. developed a facile scalable antibacterial surgical suture by using a silk-fibroin-based berberine loading system. The drug-loaded suture played a sustainable antibacterial role and exhibited satisfactory mechanical and biocompatible properties and potential for application in surgery [28]. For wound dressing, hydrogel formulations are currently gaining popularity; they facilitate healing, function as a moisturizing reservoir, and regulate inflammation and infection development. Recently, berberine was loaded into a new class of cellulose-derived biodegradable hydrogel films [29] and a gelatin/sodium alginate hydrogel [30]. The results indicated that berberine-loaded hydrogels can be used for wound dressing materials in the future. This finding is similar to that of our study, which suggests the potentiality of the mHLJD decoction against skin toxicity.

## 5. Conclusions

We developed an HPLC method to quantify the reference markers of the mHLJD decoction. The results of bioactivity analysis revealed that the mHLJD decoction extract reduced TNF-*α*-induced cytokine production and inhibited *S. aureus* growth. Our findings provide insights into potential mechanisms underlying the effects of the mHLJD decoction on EGFRI/TKI-induced skin toxicity. Thus, the mHLJD decoction represents a promising topical medicine for skin toxicity.

## Figures and Tables

**Table 1:** Linear regression of five standards of the mHLJD decoction.

Standards	*Rt* (min)	Regression equation	*R*	Linear range (*μ*g/mL)
Gentiopicrin	14.38	*y* = 6847.5*x* + 12705	0.9999	200∼3.125
Geniposide	15.03	*y* = 13401*x* + 29398	0.9999	200∼3.125
Astilbin	27.45	*y* = 8429*x* + 20999	0.9993	200∼3.125
Berberine chloride	38.93	*y* = 37137*x* + 92909	0.9991	200∼3.125
Baicalin	41.12	*y* = 9326.1*x* − 290.66	0.9992	200∼3.125

**Table 2:** The antibacterial activity of the mHLJD decoction extract.

Microorganism	Inhibition diameter (mm)^a^	Minimal inhibition concentration (MIC)^b^	
	mHLJD 5 mg	mHLJD (mg/mL)	Streptomycin (*μ*g/mL)
*S. aureus* ATCC 29213	9 ± 0	3.125	3.125
*S. aureus* ATCC 25923	10.33 ± 0.33	6.25	1.563
*S. aureus* ATCC 6538	9.33 ± 0.67	3.125	3.125
*E. coli* ATCC 8739	—	>25	3.125
*P. aeruginosa* ATCC 9027	—	>25	3.125

^a^ *n* = 3; ^b^*n* = 4.

## Data Availability

The datasets used in the current study are available from the corresponding authors on reasonable request.

## Abbreviations

CFU: Colony-forming unit
*E. coli*: *Escherichia coli*
EGFRIs: Epidermal growth factor inhibitors
GAPDH: Glyceraldehyde-3-phosphate dehydrogenase
HPLC: High-performance liquid chromatography
IL-1*β*: Interleukin-1*β*
M-CSF: Macrophage colony-stimulating factor
MH broth: Mueller–Hinton broth
mHLJD: Modified Huang-Lian-Jie-Du
MIC: Minimum inhibitory concentration
NEG: Negative control
*P. aeruginosa*: *Pseudomonas aeruginosa*
POS: Positive control
qPCR: Real-time quantitative polymerase chain reaction
*S. aureus*: *Staphylococcus aureus*
SSTIs: Skin and soft-tissue infections
TGF-*β*2: Transforming growth factor-*β*2
TKIs: Tyrosine kinase inhibitors
TNF-*α*: Tumor necrotic factor-*α*.
